# Therapeutic Effects of TianDiJingWan on the A*β*_25–35_-Induced Alzheimer's Disease Model Rats

**DOI:** 10.1155/2015/307350

**Published:** 2015-02-26

**Authors:** Zhijie Li, Qing Tong, Huifang Xu, Li Hu, Rong Zhao, Fang Zhou, Wei Pan, Li Zhou

**Affiliations:** ^1^Department of Encephalopathy, Wuhan Hospital of Traditional Chinese Medicine, Wuhan 430030, China; ^2^Department of Pharmacy, Tongji Hospital Affiliated with Tongji Medical College, Huazhong University of Science and Technology, Wuhan 430030, China

## Abstract

The main purpose of this study was to demonstrate the therapeutic effects and mechanism of TDJW, a modern Chinese medicine prescription developed based on the basic traditional Chinese medicine theory of “tonifying the kidney essence,” on the A*β*_25–35_-induced AD rats. The AD model was established by the intracerebroventricular administrations of A*β*_25–35_ into the hippocampus CA1 tissue of SD male rats. 72 rats were randomly divided into six groups: sham operation, AD model, donepezil, high TDJW group, medium TDJW group, and low TDJW group. After oral administration of TDJW, the results of Morris water maze and step-down test showed that the learning and memory abilities of AD rats were significantly improved. And biochemical measurement demonstrated that Ach and Glu in hippocampus tissues of AD rats were increased as well. Moreover, the A*β* deposits and p-Tau aggregations in hippocampus CA1 tissues of AD rats were attenuated as observed in the micrographs of immunohistochemistry study, and the results of ELISA indicated that the expressions of TNF-*α*, IL-1*β*, and IL-6 in hippocampus tissues were significantly decreased. In conclusion, the present study demonstrated that TDJW could be used as a promising therapeutic agent for the clinical applications of AD treatment in patients.

## 1. Introduction

Alzheimer's disease (AD), as a degenerative brain disease, is characterized by the sustained high nervous disorders of the brain functions and activities. Nowadays, AD has become the most general cause of dementia which is a very widespread illness in the elderly [1, 2]. However, the pathogenesis of AD is still not clarified completely at present. According to the existing research findings in treatment of AD, several options have become available for the purpose of improving AD prognosis [3]. And two major options are considered to be the most effective in treatment of AD. First of all, the acetyl choline (Ach) supplement can improve AD symptoms since the reduction of cholinergic neurons in brain directly leads to the cognitive deficiency and memory loss. So acetylcholinesterase inhibitors (AchEI) such as donepezil, rivastigmine, and galantamine that can enhance the functions of cholinergic neurons have become the most effective drugs in treatment of AD [4–6]. In addition, AD is also characterized by the overproduction and deposition of Amyloid-*β* (A*β*) in brain, and extracellular soluble A*β* oligomers are recognized as the cause of synaptic and cognitive dysfunction of AD [7, 8]. And the drug development efforts targeting the soluble A*β* oligomers have been made in the recent years [9].

As we know, traditional Chinese medicine has long been used in the clinical treatment of AD in China, because of its multicomponent and multitarget pharmacological effects with fewer side effects comparing with pure drugs [10]. In recent years, the development of anti-AD drugs from Chinese medicinal materials has drawn more and more attention from all over the world.

TianDiJingWan (TDJW), a modern Chinese medicine prescription which consists of six kinds of traditional Chinese medicine including tuber of* Gastrodia elata* Bl.,* Pheretima aspergillum *Perrier, rhizome of* Polygonatum sibiricum *Red.,* Whitmania pigra *Whitman, rhizome of* Acorus tatarinowii *Schott, and fruit of* Ligustrum lucidum *Ait., was initially developed based on the traditional Chinese medicine theory of “tonifying the kidney essence.” It has been standardized by the preparation process optimization and quality control studies (results are not shown here). And the clinical effects of TDJW in treatment of mild cognitive impairment and vascular dementia have been demonstrated by our previous clinical studies [11–13]. The main effective components of TDJW towards central nervous system were considered to be gastrodin, *β*-asarone, oleanolic acid, and polygonatum sibiricum polysaccharides according to the records from Chinese pharmacopoeia (2010 edition) and previous pharmacological studies of the traditional Chinese medicine in the prescription [14–16].

In this paper, we aimed to demonstrate the therapeutic effects and mechanism of TDJW on the A*β*_25–35_-induced AD rats. After oral administration of TDJW, the learning and memory abilities of AD rats were evaluated using the method of Morris water maze and step-down test. The glutamic acid (Glu) and acetyl choline (Ach) in the hippocampus tissues of AD rats were measured after animal behavior study, as well as the deposits of A*β* and aggregations of phosphor-Tau protein (p-Tau) in hippocampus CA1 tissues by immunohistochemistry study. Also the expressions of IL-6, IL-1*β*, and TNF-*α* in hippocampus tissues were evaluated by ELISA analysis.

## 2. Materials and Methods

### 2.1. Preparation of TDJW

The researches about the preparation process optimization and quality control of TDJW have been previously completed (results are not shown here). Briefly, according to the dosage of medicinal materials in the TDJW prescription,* Pheretima aspergillum *E. (3 g),* Whitmania pigra *Whitman (1 g), and rhizome of* Acorus tatarinowii *Schott (6 g) were procedurally cold-dried, crushed into fine powder, sifted, and blended. Then tuber of* Gastrodia elata* Bl. (10 g), rhizome of* Polygonatum sibiricum *Red. (10 g), and fruit of* Ligustrum lucidum *Ait. (10 g) were decocted 3 times with water (2 h, 1.5 h, and 1 h, resp.) and filtered separately. The filter liquor was then concentrated into paste with the relative density of 1.28–1.36 (measured at 60°C). Then the powder mixture of* Pheretima aspergillum *E.,* Whitmania pigra *Whitman, and rhizome of* Acorus tatarinowii *Schott were added into the paste and blended. After pelleting, drying, and polish, the final product of TDJW was obtained and stored properly before use.

### 2.2. Animal Model Establishment

72 SPF SD male rats weighing 200–250 g were obtained from Experimental Animal Research Center of Hubei province. All the rats were kept on the condition of 24 ± 1°C with 12 h light-dark cycle and free access to food and water. Animal experimental procedures were performed in accordance with the national regulations and guidelines and approved by the Animal Experimentation Committee of Hubei University of Chinese Medicine.

After fasting for 12 h before intracerebroventricular administration of A*β*_25–35_ (Sigma), rats were randomly assigned into six groups: sham operation group, AD model group, low dose TDJW group, medium dose TDJW group, high dose TDJW group, and donepezil group. All the rats were anesthetized with 0.45% pentobarbital sodium and fixed on the brain stereotaxic apparatus (Xi'an Wandong instrument co., LTD). The injection volume of condensate A*β*_25–35_ (1 *μ*L) was gradually injected within 5 min with a microsyringe (Shanghai Gaoge Industrial and trading co., LTD) to the hippocampus CA1 tissue according to the rat brain in stereotaxic coordinates (3.5 mm behind the bregma and 2.0 mm beside the midline; vertically insert the needle into 3.0 mm below the dura mater); the needle was then kept for 3 min and slowly withdrawn within 3 min. The sham operation group was administrated using the same method with saline. After the intracerebroventricular administration, all the rats were intramuscular administrated with penicillin (10^6^ U/mL) every other day for three consecutive times.

### 2.3. Drug Administration Procedures

A week after the animal model establishment, the rats were orally administrated as follows: AD model group and sham operation group were treated with saline, donepezil group was treated with donepezil (0.045 mg/mL, equivalent to 10 mg/day human dose), and TDJW groups were treated with low, medium, and high doses (0.1215 g/mL, 0.243 g/mL, and 0.486 g/mL, resp.) of TDJW water suspension (10 mL/kg, equivalent to 13.5 g/day, 27 g/day, and 54 g/day human dose, according to the equivalent dose ratio of 0.018 corrected by body surface area between rat and human). All the rats were treated once daily for 28 consecutive days.

### 2.4. Morris Water Maze

After drug oral administration, the Morris water maze test was carried out according to the previously described method [17]. The Morris water maze experimental system was purchased from the Institute of Materia Medica, Chinese Academy of Medical Sciences. The rat behavior was recorded by the photographic device installed above the circular pool (150 cm diameter, 50 cm height) with a 30 cm depth of water.

#### 2.4.1. Place Navigation Test

A platform was fixed on the center of the first quadrant with 2 cm below the water surface, and the water temperature was maintained at 24 ± 1°C. For each trial, the rat was allowed to stand on the platform for 10 s and then a rat was placed into a random quadrant with the head down and back to circular pool wall. The maximum swimming time was 120 s for the rat to swim to the platform. If successful within 120 s, the rat was allowed a rest period of 10 s on the platform. If unsuccessful, a score of 120 s was recorded and the rat was physically placed on the platform for a rest period of 10 s as well. Then the next trial was carried out in the same way. For place navigation test, the trials were performed twice a day for 4 consecutive days. On the 5th day, the average scores of rats from different groups were recorded as latency for further statistical analysis.

#### 2.4.2. Spatial Probe Test

After the place navigation test, the platform below the water surface was removed from the circular pool. The spatial probe test was performed the same way as place navigation test. The rat swimming path within 120 s was recorded, the time in the first quadrant and number of times crossed the former platform location were recorded as final statistics.

#### 2.4.3. Para Probe Test

One day after the spatial probe test, the para probe test was carried out in the same method as place navigation test, except that the platform was fixed on the para quadrant of former location and the rat was now placed into the para quadrant of the platform location. The trials were performed twice a day for 4 consecutive days as well. On the 5th day, the platform was removed from the circular pool, then the time in the paraquadrant and number of times crossed the platform location were recorded.

### 2.5. Step-Down Test

All the rats were allowed a 2-day rest after the Morris water maze test, and then the step-down test was performed using a step-down recorder (Huaibei Zhenghua biological instrument co., LTD) in accordance with the method previously described [18–20]. Before the experiments, the rats were placed into the reactive box for free activities. Once the power was on, the rats would receive a foot shock and then reactively look for the security platform to avoid the shock. 24 h after training sessions, the rats were physically placed on the security platform. The step-down latency and number of times received the shock within a 5 min time limit were recorded as a measurement of learning and memory performance.

### 2.6. Biochemical Measurement of Glu and Ach

After animal behavior study, 6 rats from each group were anesthetized and then executed for the purpose of Glu and Ach measurement. Both sides of hippocampus tissues were quickly removed on the ice and homogenized in 9 vol. (w/v) of saline, and the homogenate was centrifuged at 3500 rpm for 10 min. The supernatant liquor was then obtained and stored at 4°C for further biochemical measurement of Glu and Ach according to the manufacturer instructions of Glu and Ach kits (A074 and A105-1, Nanjing Jiancheng Bioengineering Institute, China).

### 2.7. Immunohistochemistry Study

The rest of 6 rats from each group were used for the immunohistochemistry study according to the method previously described [21, 22]. Briefly, 4 *μ*m hippocampus tissue paraffin section was deparaffinized with different graded xylene and alcohol. And the tissue section was treated with EDTA antigen repair buffer (G1203, Wuhan Guge biological technology co., LTD) and then washed with PBS solution (PH 7.4) every 5 min for three times on the shaker. The tissue section was placed into the 3% hydrogen peroxide solution for 20 min incubation away from light to block endogenous peroxidase and then washed with PBS solution in the same manner. Then the tissue section was incubated with A*β* IgG antibody (1 : 100, SantaCruz) and p-Tau IgG antibody (1 : 50, Abcam) diluted with 5% BSA (Sigma) overnight. After washing with PBS solution and incubating with secondary antibody for 50 min at room temperature, the tissue section was stained with DAB (DAKO) and counterstained with hematoxylin (G1004, Wuhan Guge biological technology co., LTD). Finally, the tissue section was dehydrated with different graded alcohol and xylene and then mounted. The microscopic images of hippocampus CA1 tissue sections were captured under a 10 × 20 times view, and the results of integral optical density (IOD) were calculated by analyzing the corresponding brownish yellow area in each microscopic image using Image-Pro Plus6.0 software.

### 2.8. Enzyme-Linked Immunosorbent Assay (ELISA)

After biochemical measurement of Glu and Ach, the supernatant liquor of hippocampus tissue homogenate stored at 4°C was used for the ELISA analysis. The expressions of IL-6, IL-1*β*, and TNF-*α* in hippocampus tissues of AD rats were measured in accordance with the instructions of ELISA kits (900-K86, 900-K84, and 900-K73, Neobioscience Technology, China), respectively.

### 2.9. Statistical Analysis

All the result data were expressed as means ± SEM. One-way ANOVA followed by post hoc comparisons with the least significant difference (LSD) test was used to assess the significance of differences between groups, and the criterion for statistical significance was *P* < 0.05. SPSS software 19.0 was used for the statistical analysis.

## 3. Results

### 3.1. Effects of TDJW on Learning and Memory Abilities of AD Rats

First of all, comparing the statistical data from Morris water maze test and step-down test, all the results of AD model group were significantly different (*P* < 0.01) from sham operation group except the time in the paraquadrant in paraprobe test (Figure 2(c)), indicating that the model of AD rats was successfully established by intracerebroventricular administration of A*β*_25–35_.

#### 3.1.1. Morris Water Maze

**Figure 1:**
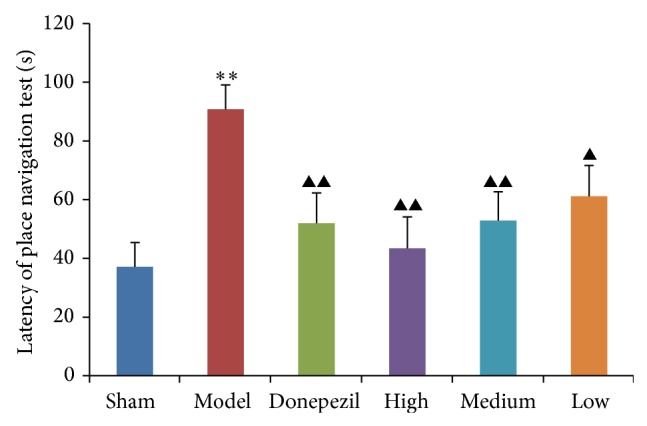
Effects of donepezil and TDJW (high, medium, low) on learning and memory improvement of AD rats in place navigation test (*n* = 12). The columns represent the mean ± SEM for latency (s) of place navigation test. ^**^ *P* < 0.01, model versus sham; ^▲^ *P* < 0.05, ^▲▲^ *P* < 0.01, donepezil or TDJW (high, medium, low) versus model.

**Figure 2:**
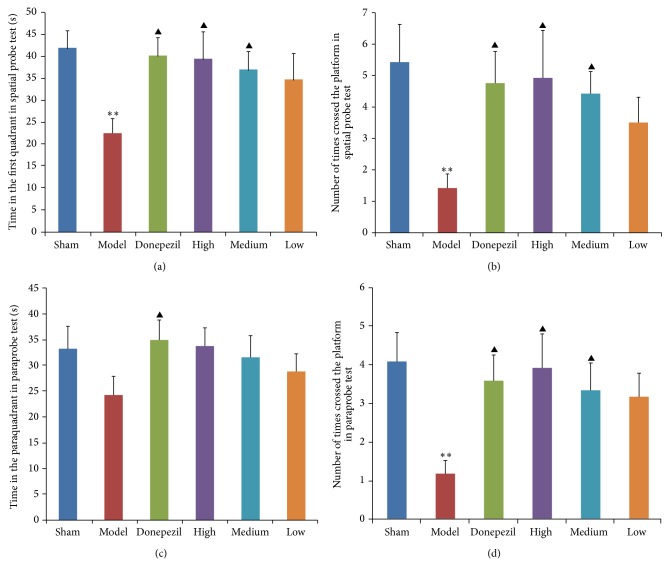
Effects of donepezil and TDJW (high, medium, low) on learning and memory improvement of AD rats (*n* = 12). The columns represent the mean ± SEM for time (s) in the first quadrant in spatial probe test (a), number of times crossed the platform in spatial probe test (b), time (s) in the paraquadrant in paraprobe test (c), and number of times crossed the platform in paraprobe test (d). ^**^ *P* < 0.01, model versus sham; ^▲^ *P* < 0.05, donepezil or TDJW (high, medium, low) versus model.

As observed in the results of place navigation test (Figure 1), donepezil had a significant effect on the latency of AD rats comparing with nontreatment of AD model group (*P* < 0.01), and different doses of TDJW had a significant improvement on the latency of AD rats (*P* < 0.05) as well. The results of spatial probe test demonstrated that the time in the first quadrant (Figure 2(a)) and number of times crossed the platform location (Figure 2(b)) of AD rats were much improved by the treatment of donepezil (*P* < 0.05). Also a significant improvement effect of TDJW (high, medium) on AD rats was observed (*P* < 0.05). As seen from the results of paraprobe test, the time in the paraquadrant and number of times crossed the platform of donepezil group had a significant difference with AD model group (*P* < 0.05). High and medium dose of TDJW significantly improved the number of times crossed the platform of AD rats (*P* < 0.05). There was no significant difference among the treatment groups of donepezil and TDJW in learning and memory improvement of AD rats. But high dose of TDJW may have a better effect than donepezil as shown in the result data such as latency of place navigation test, number of times crossed the platform in spatial probe test, and number of times crossed the platform in paraprobe test.

#### 3.1.2. Step-Down Test

**Figure 3:**
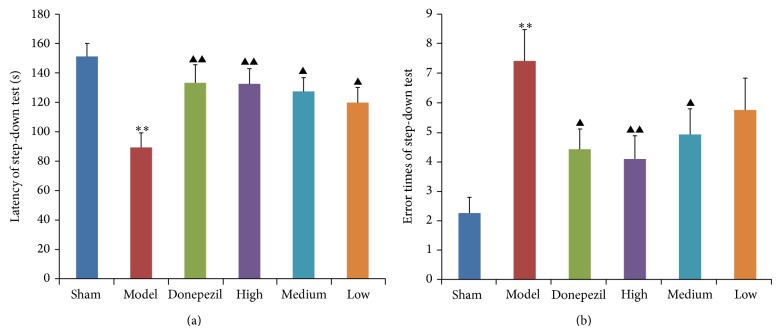
Effects of donepezil and TDJW (High, Medium, Low) on learning and memory improvement of AD rats in step-down test (*n* = 12). The columns represent the mean ± SEM for latency (s) of step-down test (a), and error times of step-down test (b). ^**^ *P* < 0.01, model versus sham; ^▲^ *P* < 0.05, ^▲▲^ *P* < 0.01, donepezil or TDJW (high, medium, low) versus model.

For the result of step-down test (Figure 3), latency and error times of AD model group were much different from those of sham operation group, demonstrating again that the model of AD rats was successfully established. Also donepezil and different doses of TDJW significantly improve the learning and memory performance of AD rats comparing with data of AD model group (*P* < 0.05), but no significant difference was observed among the treatment groups of donepezil and TDJW.

### 3.2. Biochemical Measurement of Glu and Ach

**Figure 4:**
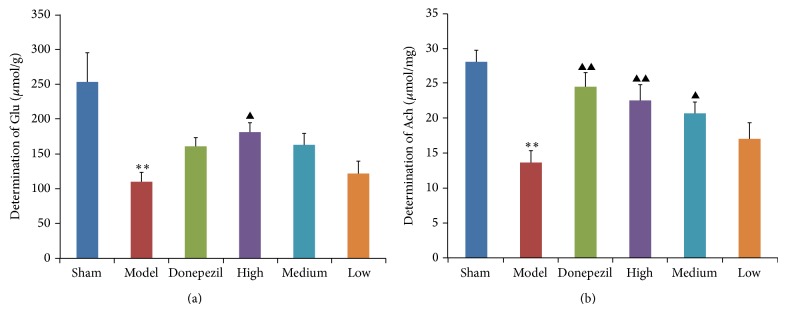
Effects of donepezil and TDJW (High, Medium, and Low) on the content of Glu (a) and Ach (b) in hippocampus tissues of AD rats resulting from biochemical measurement (*n* = 6). All the data are presented as mean ± SEM. ^**^ *P* < 0.01, model versus sham; ^▲^ *P* < 0.05, ^▲▲^ *P* < 0.01, donepezil or TDJW (High, Medium, and Low) versus model.

The biochemical measurement of Glu and Ach was conducted after Morris water maze and step-down test. As shown in the results of biochemical measurement (Figure 4), the content of Glu and Ach in hippocampus tissues of AD rats was much lower than that of sham operation group (*P* < 0.01). High dose of TDJW apparently promoted the content of Glu in hippocampus tissues of AD rats comparing with that of AD model group, while the effect of donepezil was not significant as well as medium and low dose of TDJW. As for the content of Ach, the improvement effects of donepezil group and TDJW groups were evidently observed (*P* < 0.05) except the low dose TDJW group.

### 3.3. Immunohistochemistry Study

**Figure 5:**
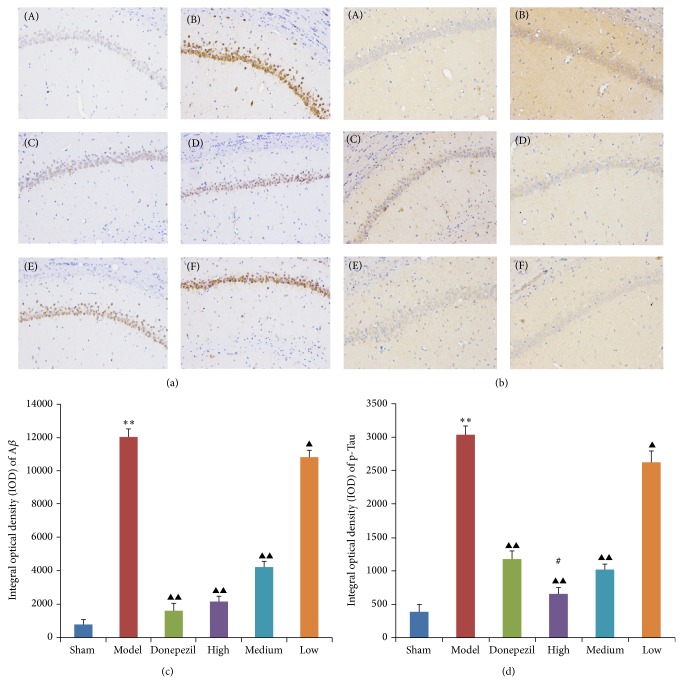
The representative immunohistochemistry micrographs of A*β* deposits (a) and p-Tau aggregations (b) in hippocampus CA1 tissues. (A) Sham operation group, (B) AD model group, (C) donepezil group, (D) high dose TDJW group, (E) medium dose TDJW group, and (F) low dose TDJW group. The IOD values of A*β* deposits (c) and p-Tau aggregations (d) were calculated by analyzing the corresponding brownish yellow area in each microscopic image using Image-Pro Plus6.0 software. All the data are presented as mean ± SEM. ^**^ *P* < 0.01, model versus sham; ^▲^ *P* < 0.05, ^▲▲^ *P* < 0.01, donepezil or TDJW (high, medium, low) versus model; ^#^ *P* < 0.05, TDJW (high, medium, low) versus donepezil.

A*β* deposits and p-Tau aggregations in hippocampus CA1 tissues of AD rats were significantly increased (*P* < 0.01) comparing with that of sham operation group. After the treatment of donepezil and TDJW, A*β* deposits and p-Tau aggregations in hippocampus CA1 tissues of AD rats were significantly attenuated comparing with that of nontreatment AD rats, especially for the treatment of high and medium dose TDJW and donepezil (*P* < 0.01). Moreover, the effect of high dose TDJW on attenuation of p-Tau aggregations was more significant than that of donepezil. All the results and microscopic images of hippocampus CA1 tissue sections are shown in Figure 5.

### 3.4. ELISA Analysis of IL-6, IL-1*β*, and TNF-*α*

**Figure 6:**
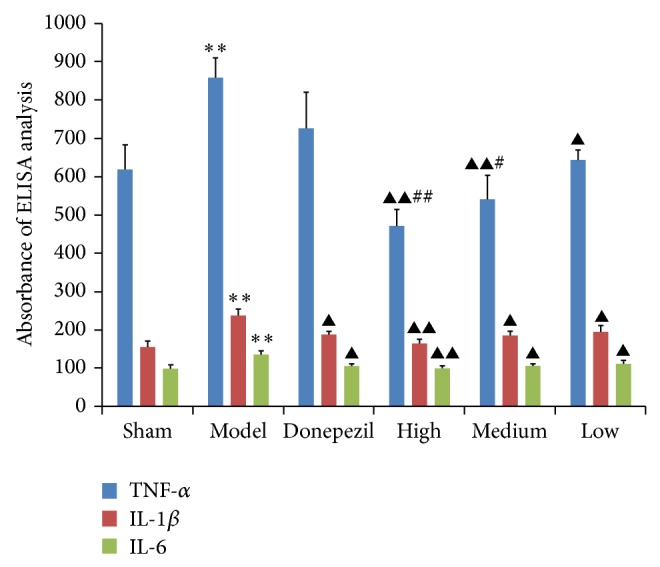
Effects of donepezil and TDJW on the expressions of TNF-*α*, IL-1*β*, and IL-6 in hippocampus tissues of AD rats resulting from ELISA analysis. All the data are presented as mean ± SEM. ^**^ *P* < 0.01, model versus sham; ^▲^ *P* < 0.05, ^▲▲^ *P* < 0.01, donepezil or TDJW (high, medium, low) versus model; ^#^ *P* < 0.05, ^##^ *P* < 0.01, TDJW (high, medium, low) versus donepezil.

As shown in the results of ELISA analysis (Figure 6), the expressions of IL-6, IL-1*β*, and TNF-*α* in hippocampus tissues of AD model group were significantly different (*P* < 0.01) from that of sham operation group. Apart from the decrease effect of donepezil on TNF-*α*, the treatment of donepezil and TDJW significantly decreased the expressions of IL-6, IL-1*β*, and TNF-*α* in hippocampus tissues of AD rats comparing with that of AD model group (*P* < 0.05). Moreover, the decrease effects of high and medium dose of TDJW were significantly different from that of donepezil.

## 4. Discussion

AD is known to be the prevalent degenerative disease of central nervous system with neuropsychiatric symptoms such as cognitive dysfunction and memory disorders. In the theory of traditional Chinese medicine, symptoms such as dementia, amnesia, muscle rigidity, and movement disorder are significantly associated with the deficiency of “kidney essence” known as “Shen Jing” in Chinese, which is believed to be one of the most important substances that determine the quality and span of life. So the basic principle of effective AD treatment is considered as “Tonifying the kidney essence,” which has been demonstrated by the clinical trial using randomized, double-blind, and parallel-controlled design [23]. Thus, as other Chinese medicinal prescriptions, TDJW was initially developed for the purpose of improving neuropsychiatric symptoms on the basic principle of tonifying the kidney essence.

In the present study, the AD model was established by the intracerebroventricular administration of A*β*_25–35_ because of its role in the AD pathogenesis such as learning and memory impairment by the inducement of inflammation and oxidative stress [24]. The results of Morris water maze and step-down test have demonstrated the success of AD model establishment since the rats in the model groups suffered the learning and memory impairment comparing with rats in sham groups. Moreover, after oral administration of TDJW, the impairment of learning and memory abilities of AD rats were significantly improved, with the effects not worse than that of donepezil.

As mentioned above, the supplement of Ach can improve the symptoms of AD. In addition, as an excitatory neurotransmitter, Glu takes a crucial part in AD pathogenesis, and the excitotoxicity and impaired metabolism of Glu had reciprocal influence on A*β* and apolipoprotein E [25–27]. The biochemical measurement demonstrated that the content of Glu in hippocampus tissues of AD rats, as well as Ach, was significantly lower than that of rats in sham groups, which was in accordance with previous research findings [28]. And the oral administration of TDJW apparently promoted the content of Ach and Glu in AD rats.

The deposits of A*β*, a core component of senile plaques, were involved in the early AD pathogenesis [29, 30], since A*β* inhibits synaptic functions which result in the deficits of early memory and synaptic degeneration and also trigger the neuronal signaling downstream responsible for p-Tau pathology of AD [10]. So increasing the clearance of A*β* deposition can significantly help to improve the AD symptoms [31]. According to the results of immunohistochemistry study, the treatment of TDJW could significantly reduce the A*β* deposits and p-Tau aggregations in hippocampus CA1 tissue of AD rats, and the reducing effect of high dose of TDJW on p-Tau aggregations was better than that of donepezil.

Unregulated inflammation and impaired inflammatory control process are highly linked to the pathogenesis of AD. IL-1, with two distinct isoforms of IL-1*α* and IL-1*β*, is considered as one of the most significant cytokines overexpressed in the pathogenesis of initial disease [32]. IL-6, similar to IL-1, also has an important role in the process of neuroinflammation associated with the pathogenesis of AD, resulting from its inhibition on other inflammatory factors such as TNF and IL-1R [33]. In addition, TNF-*α* is believed to be the major proinflammatory response regulator in brain [34]. Previous study findings showed that TNF-*α* could increase the neurotoxicity of neuronal glutamate and resulted in the cellular damage and death [35]. And the cognitive decline in patients can be improved by TNF-*α* inhibition [36]. In the present study, the different doses of TDJW treatment could significantly reduce the expressions of TNF-*α*, IL-1*β*, and IL-6 in AD rats. Moreover, the effects of high and medium dose of TDJW on TNF-*α* were better than that of donepezil.

## 5. Conclusion

In conclusion, the purpose of this study was to demonstrate the therapeutic effects and mechanism of TDJW on the A*β*_25–35_-induced AD rats. The TDJW treatment could significantly improve the learning and memory abilities of AD rats and the content of Ach and Glu in hippocampus tissues was also promoted. Also the A*β* deposits and p-Tau aggregations in hippocampus CA1 tissues of AD rats were attenuated by the treatment of TDJW. In addition, the inflammatory factors that highly associate with the pathogenesis of AD such as TNF-*α*, IL-1*β*, and IL-6 were significantly decreased in hippocampus tissues of AD rats. All these findings support the clinical applications of TDJW for the future treatment of AD patients.
